# Quality of life following completion of surgery and chemoradiation among Sri Lankan women with breast cancer

**DOI:** 10.1186/s12885-026-15991-7

**Published:** 2026-04-13

**Authors:** Harshana Wijayalathge, Karieshinie Peiris, Umesh Jayarajah, Sanjeeva Gunasekara, Don Thiwanka Wijeratne, Christopher Booth, Sanjeewa Seneviratne

**Affiliations:** 1 Department of Surgery, Faculty of Medicine, University of Colombo, P.O. Box 271, Kynsey Road, Colombo 8, Colombo, Western Province Sri Lanka; 2 National Cancer Institute, Maharagama, Sri Lanka; 3 Department of Medicine, Queen’s University, Kingston, ON Canada; 4 Division of Cancer Care and Epidemiology, Queen’s University Cancer Research Institute, Kingston, ON Canada; 5 Department of Oncology, Queen’s University, Kingston, ON Canada

**Keywords:** Quality of life, Sri Lanka, Women, Breast cancer

## Abstract

**Background:**

Breast cancer is the most common cancer globally, with rising incidence in developing countries like Sri Lanka. While advancements in treatment have improved survival rates, the post-treatment Quality of Life (QOL) of breast cancer survivors remains a critical concern. This study evaluates the QOL among Sri Lankan women with breast cancer and its association with treatment modalities.

**Methods:**

A cross-sectional study was conducted among 221 women who had undergone primary breast cancer surgery with a curative intent at least 12 months prior. Participants were recruited from the National Cancer Institute, Maharagama, between 2020 and 2022. Validated Sinhala and Tamil translations of the EORTC QLQ-C30 and QLQ-BR23 questionnaires were used to assess QOL. Demographic and clinical data were obtained from the Cancer Sri Lanka Database. Statistical analysis was performed using SPSS version 24.

**Results:**

The mean global health score was 62.6 (SD = 23.4). Functional scores for physical, role, emotional, cognitive, and social domains were satisfactory, while sexual functioning and enjoyment scores were notably low (14.5 and 18.9, respectively). Fatigue, pain, insomnia, and financial difficulties were the most prominent concerns. Radiotherapy was associated with significantly lower global health scores (*p* < 0.05), and chemotherapy was linked to a more negative future perspective (*p* < 0.01). No significant associations were found between QOL scores and the type of breast or axillary surgery.

**Conclusion:**

Sri Lankan women with treated breast cancer generally report good QOL comparable to global standards. However, sexual health remains a significant concern, particularly among older women. Fatigue, pain, and financial difficulties are notable concerns. Radiotherapy was associated with lower global health scores, while chemotherapy was observed to be associated with a more pessimistic future outlook. Addressing sexual health and financial burdens, particularly for younger patients, is essential. Future research should employ updated assessment tools and consider sociocultural factors to better understand treatment-related QOL in this population.

## Introduction

 Breast cancer is the most common cancer globally, accounting for 12% of all newly diagnosed cancers. It is also the leading cause of Disability-Adjusted Life Years (DALYs) lost worldwide, surpassing other cancer types [1]. The incidence of breast cancer is rising, particularly in developing countries, where access to high-quality cancer care remains limited. Sri Lanka, a lower-middle-income country, has witnessed a significant increase in breast cancer cases over the past two to three decades [2, 3].

Advances in surgical techniques and treatment modalities have improved survival rates for breast cancer patients [4]. In Sri Lanka, cancer care is provided through a publicly funded health system, which has expanded access to treatment and increased the number of survivors [5]. However, beyond survival metrics such as disease-free survival and overall survival, there is growing emphasis on the Quality of Life (QOL) of cancer survivors during their post-treatment years. In Sri Lanka, the QOL of breast cancer survivors is challenged by socioeconomic factors, including limited access to treatment and transportation, as well as the burden of the disease itself [6]. Consequently, post-treatment QOL has become a critical aspect of breast cancer care in the country.

Morbidity associated with breast cancer—stemming from the disease, its treatment, and psychosocial consequences—often leads to a decline in QOL. Physical symptoms common to cancer patients, as well as those specific to breast cancer, contribute to this morbidity and impair functioning. Additionally, lifestyle changes, altered body image, and sexual dysfunction further exacerbate psychosocial morbidity, adversely affecting health-related QOL. The European Organization for Research and Treatment of Cancer (EORTC) has developed validated tools, including the QLQ-C30 and QLQ-BR23, to assess QOL in cancer patients. These tools have been widely used and validated, including among Sri Lankan women with breast cancer [7–10].

Breast cancer treatment typically involves tumor resection via mastectomy or breast-conserving surgery (BCS), followed by adjuvant therapies such as chemotherapy, radiotherapy, and hormonal therapy. The choice of treatment depends on patient-specific and cancer-related factors. For early-stage breast cancer, BCS with adjuvant radiotherapy has shown survival outcomes comparable to mastectomy [11]. However, the impact of surgery on post-treatment QOL remains debated, with mixed findings across studies [12–14]. While BCS is often associated with better QOL, long-term follow-up studies suggest no significant difference between the two surgical approaches. Most of these studies originate from Western countries, raising questions about their applicability to South Asian populations. Given the sociocultural and economic differences in Sri Lanka, there is a need for local studies to explore post-treatment QOL in this context. This study aims to address this gap by evaluating post-treatment QOL and its association with treatment type among Sri Lankan women.

## Methods

A cross-sectional study was conducted among 221 women with breast cancer who had undergone primary surgery with a curative intent at least 12 months prior. Furthermore, eligibility criteria included completion of chemoradiation, absence of metastatic disease at the time of diagnosis, and no history of disease recurrence. Patients on ongoing endocrine therapy were included in the study. Patients who developed metastasis during follow-up were not excluded from the analysis. (Table 1). Participants were recruited from the National Cancer Institute, Sri Lanka (NCISL), between 2020 and 2022. Data were collected using validated Sinhala and Tamil translations of the EORTC QLQ-C30 and QLQ-BR23 questionnaires. Screening for eligibility was done through the Cancer Sri Lanka Database, a prospectively maintained database at NCISL since 2016 [15].

**Table 1:** Clinical and demographic characteristics of participants

Variable		Frequency	%
Age	< 60	115	52.0%
	> 60	94	42.5%
	(missing)	(12)	(5.4%)
Marital status	Married	165	74.7%
	Unmarried	9	4.1%
	Widowed	10	4.5%
	divorced	1	0.5%
	(missing)	36	16.3%
Breast surgery	Mastectomy	115	52.0%
	WLE	64	29.0%
	(missing)	42	19.0%
Axillary surgery	ALND	120	54.3%
	SLNB	54	24.4%
	(missing)	47	21.3%
Tumour stage	T1	45	20.4%
	T2	99	44.8%
	T3	24	10.9%
	T4	17	7.7%
	(missing)	36	16.3%
Nodal stage	N0	103	46.6%
	N1	57	25.8%
	N2	15	6.8%
	N3	10	4.5%
	(missing)	36	16.3%
Metastasis	M0	182	82.4%
	M1	3	1.4%
	(missing)	36	16.3%
Follow-up duration	Less than 2 years	67	30.3%
	More than 2 years	108	48.9%
	(missing)	46	20.8%
Chemotherapy	Yes	115	52.0%
	No	60	27.1%
	(missing)	46	20.8%
Radiotherapy	Yes	117	52.9%
	No	59	26.7%
	(missing)	45	20.4%
Hormonal therapy	Yes	103	46.6%
	No	72	32.6%
	(missing)	46	20.8%

The EORTC QLQ-C30, a generic questionnaire, includes 30 items assessing functional capacity (physical, cognitive, role, emotional, and social) and symptom severity (fatigue, pain, nausea, and vomiting). The QLQ-BR23, a breast cancer-specific questionnaire, comprises 23 items evaluating functional and symptom scales. Responses to the EORTC QLQ‑C30 and BR-23 were scored according to the guidelines provided in the EORTC Scoring Manual. Raw scores for each scale were calculated by taking the mean of the items contributing to that scale. These raw scores were then linearly transformed to a 0 −100 scale following the standard EORTC scoring procedures. Higher scores on functional scales indicate better functioning, while higher scores on symptom scales indicate worse symptoms.

All information was collected using a self-administered questionnaire. Missing questionnaire items were handled in accordance with the recommendations of the EORTC Scoring Manual. Scale scores were calculated if at least half of the items in the respective scale had been completed. In such cases, the mean of the completed items was used to compute the raw score. There were no exclusions due to inadequate responses.

Data were analyzed using SPSS version 24. Parametric tests, including independent sample t-tests, were used to compare mean scores across subgroups based on age, type of surgery, chemotherapy, and radiotherapy. Normality of the data was assessed using skewness and kurtosis (− 0.580 and − 0.036 for the global score, respectively), as well as the Kolmogorov–Smirnov and Shapiro–Wilk tests (*p* > 0.05), indicating approximate normal distribution. Analyses were exclusively univariable, and no adjustments were made for potential confounding or multiple comparisons. Age at 60 years was used as the cutoff based on previous breast cancer quality-of-life studies, enabling comparison with existing literature on older versus younger patient outcomes [16]. Ethical approval was obtained from the Ethics Review Committee of the Faculty of Medicine, University of Colombo, Sri Lanka (Ref. No. EC-17–068).

## Results

The study included 221 women with a mean age of 58 years (SD = 11.5). Of these, 45% (*n* = 94) were over 60 years old, and the mean follow-up duration was 32 months (SD = 18.5) (median follow up – 34 months, range 12–54 months). Surgical data were available for 179 patients, with 64.2% (*n* = 115) having undergone mastectomy and 69% (*n* = 120) having undergone axillary clearance.

The mean global health score was 62.6 (SD = 23.4). Functional scores for physical, role, emotional, cognitive, and social domains were 70.4, 75.9, 79.8, 82.6, and 87.9, respectively. Symptom scores for fatigue, pain, insomnia, and financial difficulties were relatively high at 33.1, 34.1, 27.9, and 33.3, respectively. Sexual functioning and enjoyment scores were notably low at 14.5 and 18.9, respectively.

Radiotherapy showed an association with lower global health scores (*p* < 0.05), while chemotherapy was associated with a more negative future perspective (*p* < 0.01). Sexual functioning scores differed significantly between age groups (*p* < 0.05), with older women reporting lower scores. No significant associations were found between QOL scores and the type of breast or axillary surgery (Table 2).

**Table 2:** Associating factors affecting QOL and functional scores

		Age		Breast surgery		Axillary surgery		Chemotherapy		Radiotherapy	
		< 60yrs	> 60yrs	Mastectomy	WLE	SLNB	ALND	Yes	No	Yes	No
Global health score	Mean	62.25	62.58	63.91	62.89	63.42	63.26	63.18	63.88	66.31	57.76
	SD	25.01	20.33	22.93	22.06	22.16	23.13	24.65	18.70	21.48	24.06
	Mean difference (*P* value)	−0.34 (0.915)		1.02 (0.772)		0.16 (0.966)		−0.70 (0.834)		8.54 (0.018)	
Physical functioning	Mean	71.71	69.10	70.02	72.59	73.71	69.50	70.76	72.00	70.34	73.10
	SD	20.28	23.33	21.38	20.46	18.87	22.02	22.44	20.53	21.54	22.14
	Mean difference (*P* value)	2.60 (0.390)		−2.56 (0.439)		4.21 (0.228)		−1.24 (0.722)		−2.76 (0.428)	
Role functioning	Mean	76.96	74.55	76.95	78.30	80.18	75.55	76.31	76.11	77.15	74.57
	SD	26.45	30.95	29.33	26.37	25.74	29.62	29.82	27.67	28.70	29.58
	Mean difference (*P* value)	2.40 (0.547)		−1.35 (0.761)		4.63 (0.326)		0.20 (0.965)		2.57 (0.579)	
Emotional functioning	Mean	78.50	81.82	79.20	80.33	84.10	77.77	77.70	80.41	79.02	78.24
	SD	24.58	22.76	25.96	22.14	20.55	25.32	25.94	23.36	24.18	26.80
	Mean difference (*P* value)	−3.31 (0.318)		−1.13 (0.768)		6.32 (0.109)		−2.71 (0.499)		0.77 (0.847)	
Cognitive functioning	Mean	82.75	81.72	82.46	81.51	82.71	81.80	80.14	83.61	82.75	78.81
	SD	21.05	22.66	21.27	20.81	19.94	21.38	22.28	21.99	20.61	24.91
	Mean difference (*P* value)	1.03 (0.734)		0.95 (0.772)		0.91 (0.791)		−3.47 (0.330)		3.94 (0.297)	
Social functioning	Mean	86.08	89.71	88.11	88.28	87.65	88.33	86.37	90.27	88.17	87.00
	SD	19.98	21.73	20.56	20.71	18.92	21.34	22.02	20.19	20.53	23.17
	Mean difference (*P* value)	−3.62 (0.211)		−0.16 (0.959)		−0.67 (0.841)		−3.90 (0.254)		1.17 (0.733)	
Body Image	Mean	81.57	82.51	79.02	83.85	81.17	80.74	79.16	85.83	80.34	84.05
	SD	23.73	24.55	26.26	20.78	26.16	24.05	24.42	22.77	23.70	24.54
	Mean difference (*P* value)	−0.93 (0.781)		−4.83 (0.179)		0.43 (0.916)		−6.67 (0.082)		−3.70 (0.337)	
Sexual functioning	Mean	20.45	7.76	12.76	16.93	16.66	13.10	14.76	13.45	15.79	11.40
	SD	21.49	17.67	18.66	20.29	22.08	17.89	18.11	23.66	19.84	20.20
	Mean difference (*P* value)	12.68 (0.000)		−4.17 (0.175)		4.56 (0.275)		1.31 (0.687)		4.39 (0.176)	
Sexual enjoyment	Mean	27.61	9.28	15.87	21.05	19.25	16.36	20.12	12.12	19.69	12.82
	SD	30.87	22.60	26.99	28.61	30.55	26.08	28.61	24.31	28.30	24.83
	Mean difference (*P* value)	18.32 (0.000)		−5.18 (0.255)		2.89 (0.551)		8.0 (0.079)		6.87 (0.136)	
Future perspective	Mean	74.26	75.26	73.04	76.56	74.69	74.16	69.85	82.22	73.50	75.70
	SD	30.08	29.44	32.11	24.97	29.62	30.08	31.21	24.13	28.88	30.84
	Mean difference (*P* value)	−0.99 (0.810)		−3.5 (0.417)		0.52 (0.915)		−12.36 (0.008)		−2.20 (0.641)	

## Discussion

This study evaluated post-treatment QOL among Sri Lankan women with breast cancer using validated tools. Overall, participants reported satisfactory QOL, with good global health and functional scores. However, sexual functioning and enjoyment were notably poor, highlighting an important area for further attention. Fatigue, pain, insomnia, and financial difficulties were identified as most prominent symptom burdens.

The mean global health score in this study (62.6) was comparable to global reference values (61.3) [17] and findings from previous local studies [9, 16]. In contrast to some prior studies, age was not significantly associated with overall QOL in our cohort [18, 19], although older women reported lower sexual functioning scores.

Sexual functioning scores were low across the cohort, with significantly lower scores observed among older participants. While the underlying reasons for this were not directly assessed in this study, this finding is consistent with prior literature reporting reduced sexual functioning among breast cancer survivors [20, 21]. These findings highlight the need for greater attention to sexual health in survivorship care.

Although overall symptom scores were relatively low, fatigue, pain, insomnia, and financial difficulties were the most prominent concerns reported by participants. These findings are in keeping with previous studies identifying these domains as key contributors to reduced QOL among breast cancer survivors [22].

Radiotherapy was associated with lower global health scores; this finding is consistent with previously reported associations [23, 24]. Our findings indicate that participants who received chemotherapy reported a more negative future outlook. Persistent side effects and long-term complications of chemotherapy may contribute to these perceptions, affecting both physical functioning and psychosocial well-being [25]. However, as this study is cross-sectional, these relationships should be interpreted as associations rather than causal effects.

No significant associations were observed between QOL scores and the type of breast or axillary surgery. This is consistent with some previous reports suggesting comparable QOL outcomes across different surgical approaches [14, 26, 27].

This study has several limitations. It was conducted at a single tertiary care center and used a cross-sectional design, which limits causal interpretation. Furthermore, this study utilized an older QOL tool (QLQ-BR23); future studies should consider using the updated QLQ-BR45 to provide a more comprehensive assessment of QOL in the context of evolving treatment modalities [28]. Sociocultural factors influencing QOL that may be unique in Sri Lanka needs to be explored which will help develop more effective interventions to improve QOL among women with breast cancer in Sri Lanka. The small proportion of missing data were primarily due to incomplete responses in the self-administered questionnaires. Where possible, missing information was retrieved through follow-up using the contact details provided. For analysis, available-case analysis was performed, and no imputation methods were applied.

## Conclusion

Sri Lankan women with treated breast cancer generally report good QOL, comparable to global standards. Overall, participants reported satisfactory QOL, with good global health and functional scores. However, sexual health remains a significant concern, particularly among older women. Fatigue, pain, and financial difficulties are notable symptom burdens. Radiotherapy was associated with lower global health scores, while chemotherapy was observed to be associated with a more pessimistic future outlook. Addressing sexual health and financial burdens, particularly for younger patients, is recognized as a priority. Future research should employ updated assessment tools and consider sociocultural factors to better understand treatment-related QOL in this population.

## Data Availability

The datasets used and/or analysed during the current study are available from the corresponding author on reasonable request.

## Abbreviations

QOL: Quality of Life
DALY: Disability-Adjusted Life Years
NCISL: National Cancer Institute, Sri Lanka
WLE: Wide local excision
SLNB: Sentinel lymph node biopsy
ALND: Axillary lymph node dissection
